# Role of myeloid-derived suppressor cells in the formation of pre-metastatic niche

**DOI:** 10.3389/fonc.2022.975261

**Published:** 2022-09-27

**Authors:** Guoqi Ya, Weihong Ren, Rui Qin, Jiao He, Shuo Zhao

**Affiliations:** ^1^ The First Clinical Medical Institute, Henan University of Chinese Medicine, Zhengzhou, China; ^2^ Department of Laboratory Medicine, The First Affiliated Hospital of Henan University of Chinese Medicine, Zhengzhou, China

**Keywords:** myeloid-derived suppressor cells, pre-metastatic niche, circulating tumor cells, immunosuppression, targeted therapy

## Abstract

Metastasis is a complex process, which depends on the interaction between tumor cells and host organs. Driven by the primary tumor, the host organ will establish an environment suitable for the growth of tumor cells before their arrival, which is called the pre-metastasis niche. The formation of pre-metastasis niche requires the participation of a variety of cells, in which myeloid-derived suppressor cells play a very important role. They reach the host organ before the tumor cells, and promote the establishment of the pre-metastasis niche by influencing immunosuppression, vascular leakage, extracellular matrix remodeling, angiogenesis and so on. In this article, we introduced the formation of the pre-metastasis niche and discussed the important role of myeloid-derived suppressor cells. In addition, this paper also emphasized the targeting of myeloid-derived suppressor cells as a therapeutic strategy to inhibit the formation of pre-metastasis niche, which provided a research idea for curbing tumor metastasis.

## Introduction

According to statistics, the number of cancer patients in the world has exceeded 19.3 million in 2020, with nearly 10 million cumulative deaths (1). For decades, research of cancer biologist on cancer metastasis has mainly focused on the causes of carcinogenic transformation. In recent years, due to the high mortality related to metastasis, cancer biologists are forced to turn their research to the process of tumor metastasis. After massive research, it is found that the host organ has made a series of preparations for the arrival of tumor cells before tumor metastasis, such as recruiting immune cells, remodeling matrix, generating new blood vessels, etc. These changes create a favorable environment for the colonization and dissemination of tumor cells, that is, the pre-metastasis niche (PMN) (2).

Myeloid-derived suppressor cells (MDSCs) are a group of heterogeneous cells from bone marrow with strong immunosuppressive activity. In the 1970s, MDSCs were first found in a cancer patient. After years of research, it was found that these cells are related to the occurrence and development of inflammation, chronic infection, autoimmune diseases, cancer and other diseases. In order to better describe the origin and function of such cells, researchers suggested that this group of cells be named MDSCs (3). MDSCs play an important role in promoting PMN and maintaining tumor metastasis due to their immunosuppressive function. Increasing evidence that MDSCs contributed to cancer metastasis can bring us the opportunity to develop new therapeutic approaches and enhance patient’s survival. Therefore, understanding how MDSCs can promote and maintain metastasis has laid a foundation for studying some unsolved problems and developing clinical applications. In this article, we introduced the phenotype of MDSCs and the process of PMN formation, and mainly discussed the important contribution of MSDCs in the induction of the PMN and summarized the clinical application of MDSCs targeted therapy.

## Origin and phenotype of MDSCs

MDSCs are derived from hematopoietic stem cells (HSCs) (4). Under physiological conditions, HSCs differentiate into common myeloid progenitors (CMPs) and then differentiate into granulocyte-monocyte progenitors (GMPs). Immature myeloid cells (IMCs) such as CMPs and GMPs migrate to peripheral organs and differentiate into mature granulocytes, dendritic cells (DCs) and macrophages, and enter the corresponding tissues and organs to play a normal immune response. Under pathological conditions, such as trauma, infection and cancer, inflammatory factors will inhibit the differentiation of IMCs into mature myeloid cells, make it stay in various differentiation stages, and finally form MDSCs with immunosuppressive function (5).

MDSCs can be divided into polymorphonuclear or granulocytic MDSCs (PMN/G-MDSCs) and monocytic MDSCs (M-MDSCs) according to their phenotypic and morphological characteristics (6). The phenotypes of MDSCs in different species are different. Due to the high expression of myeloid differentiation antigens Gr-1 and CD11b in mice, the phenotype of M-MDSCs in mice is defined as CD11b+Ly6G−Ly6Chigh, while the phenotype of PMN-MDSCs is defined as CD11b+Ly6G+Ly6Clow (7). In 2017, Goldmann (8) and others found a new subgroup of MDSCs in mice infected with staphylococcus aureus and named it Eo-MDSCs. Because Eo-MDSCs resemble eosinophil morphologically and express the eosinophil marker Syglec-F, their phenotype is defined as CD11b+SyglecF+CCR3lowIL-5RαlowSSC-Ahigh.

Compared with mouse MDSCs, human MDSCs lack the Gr-1, and mainly express CD11b, CD33, HLA-DR, Lin (including CD3, CD14, CD15, CD19, AND CD56) (6, 9). Due to the complex classification of human MDSCs, it is widely accepted that M-MDSCs are defined as CD11b+CD14+HLA-DR−CD15− and PMN-MDSCs are defined as CD11b+CD14−CD15+ or CD11b+CD14−CD66b+ (9). In addition to the well-known PMN-MDSCs and M-MDSCs, there is another phenotype of human MDSCs. This class of MDSCs is considered immature due to the absence of granulocyte or monocyte markers and is therefore described as early MDSCs, whose phenotype is defined as Lin−HLA-DR−CD33+ (10). In addition, a subpopulation of MDSCs with a fibrocytic phenotype, known as fibrocytic MDSCs (F-MDSCs), was identified in pediatric metastatic sarcoma patients (11) and healthy neonatal cord blood (12). They defined the F-MDSCs phenotype as CD11b−CD11c−CD33+IL-4Rα+ (11, 13) (**Table 1**).

**Table 1:** Phenotypes of MDSCs.

Subsets	Phenotypes	References
PMN-MDSCs(Human)	CD11b+CD14−CD15+/CD11b+CD14−CD66b+	Pawelec (9)
	CD15+CD11b+CD33+HLA-DR−Lin−	Gabrilovich (3)
M-MDSCs(Human)	CD11b+CD14+HLA-DR−CD15−	Pawelec (9)
	CD14+CD11b+CD33+HLA-DR−Lin−	Gabrilovich (3)
e-MDSCs(Human)	Lin−HLA-DR−CD33+	Gabrilovich (10)
F-MDSCs(Human)	CD11b−CD11c−CD33+IL-4Rα+	Mazza (12)
PMN-MDSCs(Mouse)	CD11b+Ly6G+Ly6Clow	Youn (7)
M-MDSCs(Mouse)	CD11b+Ly6G−Ly6Chigh	Youn (7)
Eo-MDSCs(Mouse)	CD11b+SyglecF+CCR3lowIL-5RαlowSSC-Ahigh	Goldmann (8)

## PMN formation

Tumor metastasis is a multi-step process, including invasion, circulation, exosmosis, colonization and so on (14–16). Christine Chaffer (17) and others summarized this complex cascade reaction into two stages. The first stage is the physical migration of cancer cells from the primary tumor to the host organ, the second stage is when cancer cells colonize the host organ and develop into metastases. However, the host organs that form metastases are not randomly selected, but determined by the organotropism of the primary tumor. The “seed and soil” hypothesis proposed by Steven Paget (18) in 1989 explained organotropism during tumor metastasis, which was also supported by Fidler (19, 20), who showed that the outcome of metastasis depended on the characteristics of tumor cells and host organs. Later researchers also came to light by realizing that breast cancer was found to predispose to bone, liver, brain, and lung metastases, colorectal cancers predominantly develop liver metastases, and tumors originating in the breast, bladder, colon, kidney, head and neck and melanoma have a tendency to cause metastases in the lung (21). In 2005, Kaplan found that myeloid hematopoietic progenitors expressing vascular endothelial growth factor receptor 1 (VEGFR1) arrived at the host organ before tumor cells and changed the microenvironment there (2). Based on these results, they proposed the concept of “premetastatic microenvironment”, in which primary tumors induce host organs to create a microenvironment that supports tumor cell metastasis. The presence of the PMN more strongly explains organ tropism during tumor metastasis and illustrates the importance of the interaction between tumor cells and host organs.

**Figure 1:**
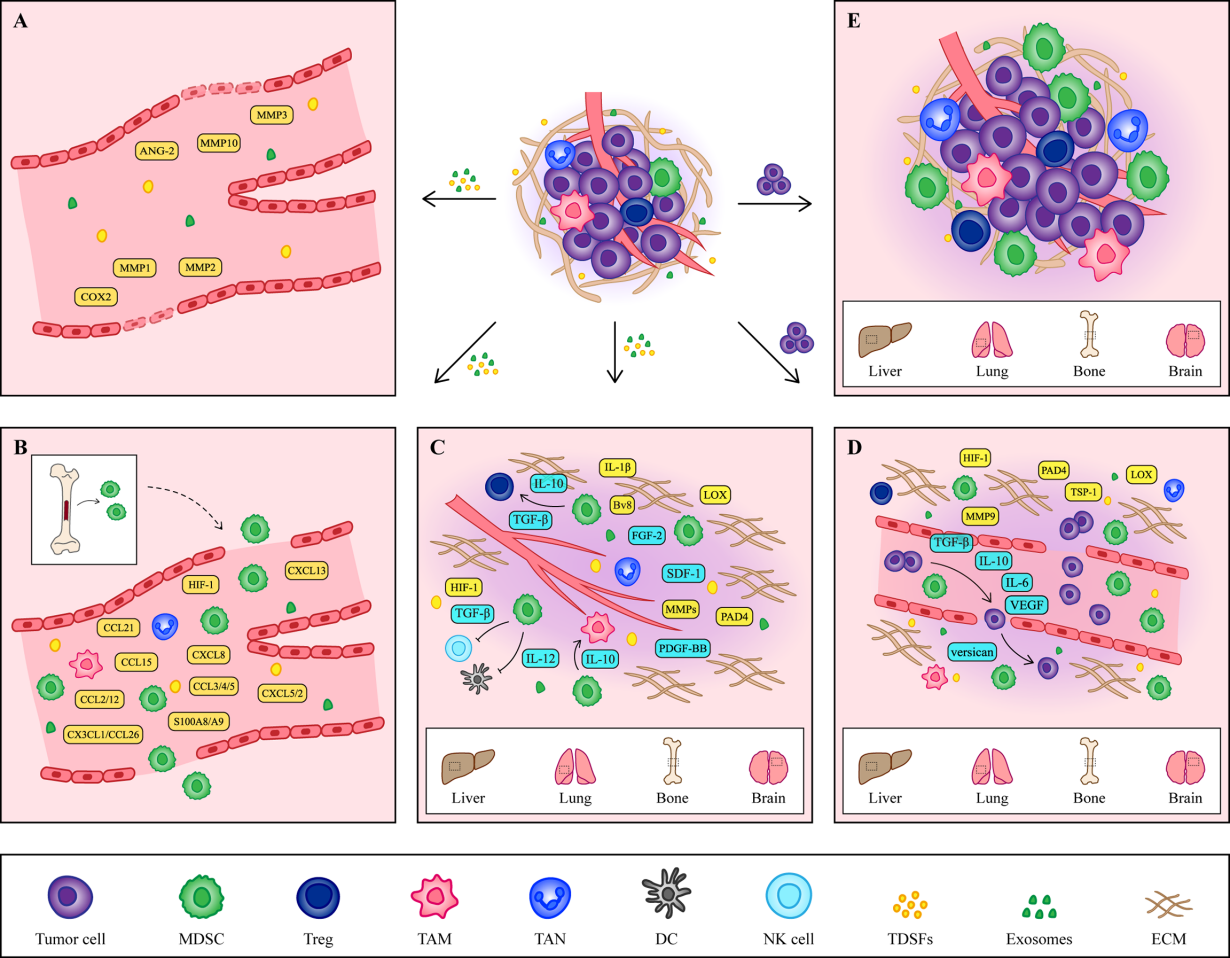
The formation of PMN. **(A)** Vascular leakage is the initial marker of PMN formation. Cytokines such as COX2, MMP1 and MMP2 secreted by primary tumors increase the permeability of endothelial cells and help the exudation of tumor cells. **(B)** Under the induction of hypoxic microenvironment and TDSFs, MDSCs and other immune cells are recruited to PMN, creating an excellent immune microenvironment for tumor cells. **(C)** At the same time, the ECM in the host organ began to change, providing a good environment for the growth of tumor cells. The accumulation of fibronectin and the crosslinking of collagen I provide a platform for the adhesion of MDSCs. At the same time, MMPs promote angiogenesis and contribute to tumor cells intrusion. In addition, some cytokines secreted by MDSCs also contribute to the remodeling of ECM. **(D)** Tumor cells arriving at the host organ will enter a dormant state, which is regulated by cytokines secreted by tumor cells and MDSCs. When tumor cells wake up, they continue to grow. **(E)** The formation of the PMN is a comprehensive result of vascular leakage, MDSCs recruitment and ECM remodeling. The tumor cells shed from the primary site and colonized the host organ, eventually forming a distant metastasis.

The formation of the PMN is the result of the joint action of multiple cytokines or cellular components including tumor-derived secretory factors (TDSFs), exosomes, bone marrow-derived cells (BMDCs) and extracellular matrix (ECM) (22) (**Figure 1**). Since PMN formation is a complex dynamic change, this paper mainly summarizes it from the following three aspects.

### Vascular leakiness

In the process of tumor metastasis, cancer stem cells (CSCs) with self-replication and differentiation ability first lose the cell-cell adhesion ability and detach from the primary site, invade the surrounding tissues and enter lymphatic vessels and blood vessels. In this process, CSCs undergo epithelial mesenchymal transition (EMT) to become circulating tumor cells (CTCs) (14). CTCs entering the blood vessels are surrounded by platelets, clotting factors and fibrin, preventing the killing effect of NK cells. Meanwhile, selectin and integrin secreted by platelets mediate the adhesion and adhesion of CTCs to vascular endothelial cells, preparing for CTCs extravasation (23, 24). Therefore, the changes of vascular endothelial cells are the key markers for the initial establishment of the PMN.

The current study revealed a relationship between vascular stability and TDSFs. Among them, cyclooxygenase 2 (COX2), matrix metalloproteinase 1 (MMP1) and matrix metalloproteinase 2 (MMP2) secreted by primary tumors were found to affect vascular integrity by changing the morphology of endothelial cells and improving vascular permeability in the lung metastasis model of breast cancer mice (25). In a melanoma mouse model of lung metastasis, primary tumors disrupt pulmonary vascular stability by upregulation of angiopoietin-2 (ANG-2), MMP3, and MMP10 in pulmonary blood vessels (26).

In addition to TDSFs, tumor-derived exosomes are also involved in regulating intracellular stability. Human colon cancer cell derived exosomal miR-25-3p increases vascular permeability in mouse lung and liver metastasis models by regulating the expression of VEGF2 and zonula occludens 1 (ZO-1), occludin and Claudin5 in endothelial cells (27). MiR-103, miR-638, miR-663a, miR-3648 and miR-4258 secreted by HCC attenuated the integrity of endothelial cells by down-regulating the expression of VE-cadherin and ZO-1 (28, 29). MiR-3157-3p regulates endothelial cell permeability and promotes angiogenesis by regulating the expression of vascular endothelial growth factor (VEGF), MMP2, MMP9 and occludin in Non-small cell lung carcinoma (30). Changes in the stability and integrity of vascular endothelial cells prepare for CTCs extravasation, so timely prevention of endothelial cell changes in the early stage of cancer can help block the formation of the PMN and prevent tumor metastasis.

### MDSCs recruitment

In fact, primary tumors shed tens of thousands of cancer cells into the bloodstream each day, but because of oxidative stress, shear forces, and immune system attacks, only a few make it into the host organs to form metastases (31, 32). Therefore, the host organ needs to establish a good immune microenvironment to protect CTCs from elimination by NK cells, CD4+T cells and CD8+T cells. Under the action of TDSFs, BMDCs and immune cells such as regulatory T cells (Tregs), tumor associated macrophages (TAMs), and tumor associated neutrophils (TANs) are recruited to host organs to help establish the immune microenvironment (33, 34). Among them, BMDCs are the main participating cells, which protect CTCs by secreting a large number of cell components, and the MDSCs subgroup that constitutes BMDCs is considered to be the most critical cell group (35).

As an important marker of PMN initiation and evolution, the recruitment of MDSCs is a multi-step process, which is mainly regulated by tumor microenvironment and TDSFs. The hypoxic environment in the tumor microenvironment is considered to be an important factor in inducing the recruitment and activation of MDSCs. Sceneay showed that primary tumor hypoxia provides cytokines and growth factors capable of creating a premetastatic niche through recruitment of MDSCs (36). Then more studies found that hypoxia inducible factor 1 (HIF-1) induced the migration of MDSCs into PMN by up regulating the expression of CCL26 (37), programmed death-ligand 1 (PD-L1) (38) and ectonucleoside triphosphate diphosphohydrolase 2 in tumor cells (39). Among TDSFs, chemokines are the main components regulating the recruitment of MDSCs (**Table 2**). In addition to chemokines, other TDSFs, such as S100A8/A9, are also involved in the recruitment of MDSCs. S100A8/A9 from breast cancer cells promotes the migration and accumulation of MDSCs through the NF-κB signaling pathway. However, S100A8/A9 not only comes from tumor cells, but also can be synthesized and secreted by MDSCs (60). Therefore, S100A8/A9 may provide an autocrine pathway for PMN to recruit MDSCs.

**Table 2:** Major chemokines involved in regulating MDSCs recruitment.

Chemokines	Receptors	Source	MDSC subsets	References
CCL2	CCR2	Pancreatic ductal adenocarcinoma	M-MDSCs	Gu (40)
		Lung cancer	MDSCs	Hartwig (41)
		Gastric cancer	MDSCs	Zhou (42)
CCL3/4/5	CCR5	Ret transgenic melanoma mice	MDSCs	Blattner (43)
		Prostate cancer	PMN-MDSCs	Hawila (44)
		Breast cancer	MDSCs	Luo (45)
CCL15	CCR1	Colorectal cancer	MDSCs	Inamoto (46)
CCL26	CX3CR1	Hepatocarcinoma cell cancer	MDSCs	Chiu (38)
CX3CL1	CX3CR1	Lewis lung cancer and spindle cell tumour	M-MDSCs	Okuma (47)
CXCL1	CXCR2	Colorectal cancer	MDSCs	Wang (48)
		Hepatocarcinoma cell cancer	MDSCs	Xia (49)
		Hepatocarcinoma cell cancer	MDSCs	He (50)
CXCL2	CXCR2	Bladder cancer	MDSCs	Zhang (51)
		Ovarian cancer	PMN-MDSCs	Taki (52)
		Colorectal cancer	MDSCs	Chen (53)
CXCL5	CXCR2	Renal cell carcinoma	PMN-MDSCs	Najjar (54)
		Bladder cancer	MDSCs	Lin (55)
		Gastric cancer	MDSCs	Zhou (42)
		Breast cancer	MDSCs	Yu (56)
CXCL8	CXCR1/2	Non-small cell lung cancer	MDSCs	Zadian (57)
		Esophageal squamous cell carcinoma	MDSCs	Yue (58)
CXCL12	CXCR4	Gastric cancer	MDSCs	Zhou (42)
		Colorectal cancer	MDSCs	Yu (59)

### ECM remodeling

Since the host organ and the primary site of the tumor are not the same in tissue and structure, in order to establish a PMN suitable for the growth and colonization of CTCs, the ECM structure in the host organ will change, promoting tumor cell migration and invasion into the stromal tissue. During the formation of the PMN, primary tumors remodel ECM by inducing stromal cells to deposit new ECM components or modify old ECM components by TDSFs.

As we all know, Lysyl oxidase (LOX) and MMPs secreted by primary tumors play an important role in ECM remodeling. LOX increases the stiffness of ECM by catalyzing collagen and elastin crosslinking. At the same time, LOX can also promote the recruitment of BMDCs and drive the formation of osteolytic lesions, leading to bone metastasis (61–63). While MMPs play a role in degrading ECM and inducing angiogenesis (64). In addition, recent studies have found that tumor-produced peptidylarginine deiminase 4 (PAD4) also helps to remodel ECM. PAD4 produced by colorectal cancer affects the citrullination of ECM, which promotes EMT and eventually leads to tumor metastasis to the liver. Preventing citrullination of ECM may create an adverse microenvironment for cancer cell growth (65). Therefore, PAD4 and citrullination may be an effective target for the treatment of liver metastases.

CTCs entering the host organ either apoptosis or dormant until all environments become suitable for CTCs to awaken (66). A growing body of research has shown that the composition of ECMs and their effect on adhesion signaling not only provides a favorable environment for the growth of CTCs, but also regulates the dormancy and awakening of tumor cells (67). For example, endothelial-derived thrombospondin-1 (TSP-1) (68) and osteopontin secreted by osteoblasts (69), as well as matrigel, commonly used for cultured cells in vitro (70), can induce tumor cell dormancy. Recently, studies have found that dormant tumor cells assemble a type III collagen-enriched ECM niche. Type III collagen triggers STAT1 activation and nuclear translocation to regulate COL3A1 expression by binding to discoidin domain receptor 1 (DDR1). The increase of COL3A1 expression in turn remodeled the ECM by increasing the curl of the ECM and brought the cells into a dormant state maintained by DDR1 binding (71). Thus, the manipulation of these mechanisms could serve as a barrier to metastasis through disseminated tumor cell dormancy induction. While the awakening of tumor cells is associated with neutrophil elastase and MMP9, which induce the proliferation of dormant CTCs by exposing laminin epitopes associated with CTCs (72). Given the central role of the ECM in tumor metastasis, altering the structure, function, and biological properties of the ECM and modulating adhesion signaling promises to reduce tumor invasion and prevent tumor metastasis, providing new therapeutic strategies for cancer patients.

After a series of changes, the PMN eventually become mature, and subsequently more and more tumor cells migrate to this site to grow, proliferate, and thus form tumor metastases. Although this article divides the process of PMN formation into three main parts, the establishment and eventual progression of PMN into metastases is an integral and dynamic process, and therefore each of these changes is critical and does not act as an independent contributing factor.

## Role of MDSCs in PMN formation

Since metastasis is intrinsically an inefficient process, most cancer cells are unable to metastasize to host organs. Primary tumors therefore need to develop strategies that can both suppress immune responses and alter the tissue framework when inducing PMN formation, providing the most effective aid to tumor metastasis. In this strategy, MDSCs with immunosuppressive effect are the best partners for tumor cells, which not only create a favorable growth environment for tumor cells but also provide much help for their metastasis. The important roles played by MDSCs are mainly reflected in two aspects: immunological effects and non-immunological effects. On the one hand, the immunosuppressive effect of MDSCs promotes tumor cell survival. On the other hand, MDSCs promote tumor cell metastasis by promoting EMT and mesenchymal epithelial transition (MET), protecting CTCs and promoting their extravasation, as well as inducing angiogenesis (73, 74).

### Immunological effects of MDSCs

**Figure 2:**
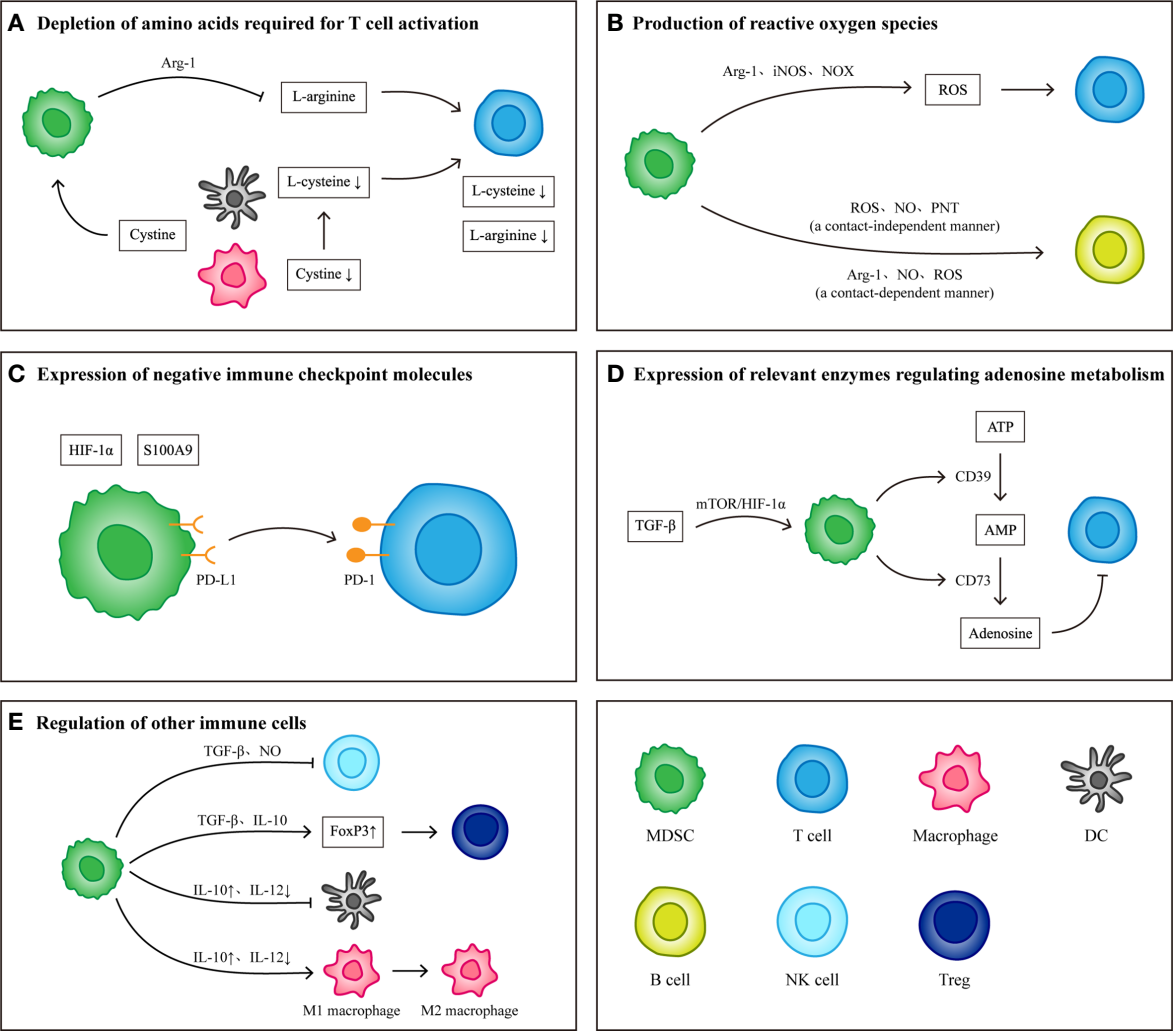
Immunological effects of MDSCs. **(A)** MDSCs consume L-arginine and L-cysteine required for T cell activation by expressing Arg-1 and ingesting cystine. **(B)** MDSCs produce reactive oxygen species by expressing Arg-1, iNOS and NOX. Different components of reactive oxygen species have inhibitory effects on T cells and B cells. **(C)** Under the induction of S100A9 and HIF-1α, the expression levels of PD-L1 on MDSCs increased. High levels of PD-L1 combined with PD-1 on T cells induced T cell apoptosis. **(D)** TGF-β Induce MDSCs to produce CD39 and CD73, which can induce the conversion of ATP to adenosine. The presence of adenosine inhibits the activation and proliferation of T cells. **(E)** In addition to inhibiting T cells, MDSCs can also regulate other immune cells to protect tumor cells and establish immune microenvironment. MDSCs inhibit the killing effect of NK cells by producing TGF-β and NO) they weakened the antigen-presenting function of DCs and macrophages by producing IL-10, and transformed M1 macrophages into M2 macrophages) they also secrete TGF-β and IL-10 up regulates the expression of FoxP3 in Tregs and induce the production of FoxP3^+^ Tregs.

The immunosuppressive effects exerted by MDSCs include specific immunity and nonspecific immunity, both of which exert corresponding immunosuppressive effects by suppressing T cells. During the formation of the PMN, MDSCs, as the main cell group involved in the establishment of the immune system, play a key role in the survival of CTCs and the establishment and maintenance of an immunosuppressive microenvironment in host organs. The main mechanisms of action include depletion of amino acids required for T cell activation, production of reactive oxygen species (ROS), expression of negative immune checkpoint molecules, expression of relevant enzymes regulating adenosine metabolism and regulation of other immune cells (**Figure 2**).

#### Depletion of amino acids required for T cell activation

L-arginine and L-cysteine are considered essential amino acids for T cell activation (75, 76). Deficiency in L-arginine leads to a block in the synthesis of CD3ζ in the T cell receptor (TCR), which plays a central role in initiating the signal transduction cascade leading to T cell activation, and therefore downregulation of CD3ζ affects T cell activation and function (77). Concomitant L-arginine deficiency also causes T cell cycle arrest in G0-G1 phase by upregulating cyclin D3 and cyclin-dependent kinase 4 (78). Meanwhile, the participation of L-cysteine is also required during T cell activation. Because T cells lack cystathionase and an intact cystine transporter, it is unable to produce L-cysteine or convert intracellular methionine to L-cysteine. Therefore, the uptake of L-cysteine by T cells depends on the supply of other cells (76).

MDSCs were first shown by Bronte in 2003 to express highly active arginase-1 (Arg-1), and MDSCs inhibit T cell proliferation in an Arg-1-dependent mechanism (79). Subsequently, researchers in multiple studies have confirmed the mechanism by which MDSCs break down L-arginine required by T cells to inhibit T cell proliferation and activation by expressing Arg-1, and showed that Arg-1 can serve as a breakthrough point to block the suppressive effect of MDSCs (80–82). However, it was found that MDSCs mediated T cell suppression does not have to be Arg-1 dependent. In 2018, Bian found that either Arg-1-expressing MDSCs or Arg-1-deficient MDSCs exhibited a strong inhibitory effect on the proliferation of T cells, and found that MDSCs inhibited the proliferation of T cells required direct contact between cells (83). Clearly, further studies are needed to investigate the remaining question regarding the role of arginase-1 MDSC function, if not for mediating the inhibition of T cells.

In addition, MDSCs can competitively take up cystine with macrophages and DCs, which renders macrophages and DCs unable to take up cystine and provide its reduction to L-cysteine to T cells, resulting in limited T cell activation (76).

#### Production of reactive oxygen species

MDSCs generate ROS, including superoxide anion, H2O2, hydroxyl radical, NO, and others, by expressing Arg-1, inducible nitric oxide synthase (iNOS), and NADPH oxidases (NOX) (84). Although ROS are toxic to most cells, MDSC survive despite their elevated content and release of ROS (85). H2O2 produced by MDSC restricts the activation of T cells by reducing the expression of T cell CD3ζ (86), while NO blocks the proliferation of T cells by inhibiting IL-2 signal (87), and also inhibits NK-cell FcR-mediated functions including antibody-dependent cellular cytotoxicity, cytokine production, and signal transduction in a contact-independent manner (88). In addition, MDSCs can promote peroxynitrite (PNT) production by expressing iNOS, which in turn can inhibit antigen-specific T cell responses by nitrating the TCR so that CD8+ T cells are unable to bind to and respond to peptide major histocompatibility complexs (89, 90).

The production of ROS by MDSCs can inhibit not only T cells and NK cells but also B cells. Rastad have experimentally demonstrated the ability of M-MDSCs to suppress B cells in a cell contact independent manner through the production of ROS, NO, PNT and others in murine AIDS models (91). And then Stiff demonstrated that PMN-MDSCs could inhibit B cell proliferation in a cell contact dependent manner by producing Arg-1, NO, ROS (88). Moreover, multiple studies have reported that the regulation of Tregs by macrophages involves ROS generation (92, 93), and that Tregs are less able to resist oxidative stress in the tumor microenvironment and can undergo apoptosis under the induction of ROS compared with T cells. These apoptotic Tregs mediated immunosuppression via adenosine and A2A pathways (94). Although the interaction of ROS in MDSCs and Tregs is unclear, Tregs mediated suppression has been demonstrated to benefit from oxidative stress conditions. Therefore, it can be speculated that ROS produced by MDSCs may induce the suppressive effects of Tregs.

#### Expression of negative immune checkpoint molecules

Immune checkpoint molecules are key molecules that regulate the balance between activation and inhibition of immune responses. In the physiological state, immune checkpoint molecules block inhibitory signals for T cell activation and generate potent antitumor responses, but in tumors, negative immune checkpoint molecules mediate tumor immune escape (95). PD-L1 is a common negative immune checkpoint molecule that induces T cell apoptosis by binding to programmed death-1 (PD-1) receptors on T cells (96). PD-L1 is highly expressed in tumor derived MDSCs, especially in PMN-MDSCs (97, 98). The levels of PD-L1 expression in MDSCs was significantly increased under hypoxia, and blockade of PD-L1 at this time downregulated the immunosuppressive effect of MDSCs on T cells, suggesting that MDSCs could suppress T cells by expressing PD-L1 and that HIF-1α contributes to the induction of PD-L1 expression (37). Later, it was shown that S100A9 secreted by MDSCs also induces PD-L1 expression and that the mechanism is related to aberrant activation of the c-Myc (99). In conclusion, low MDSCs levels before tumor treatment are positively correlated with patient survival, and therefore, MDSCs can be considered as a predictive biomarker when treating with immune checkpoint inhibitors.

#### Expression of relevant enzymes regulating adenosine metabolism

Adenosine is an important signaling molecule in regulating the body’s immune response. Ectonucleoside triphosphate diphosphohydrolase 1 (also known as CD39) catalyzes the dephosphorylation of ATP to generate amp, which is then dephosphorylated to generate adenosine under the catalysis of ecto-5’-nucleotidase (also known as CD73). Ultimately, adenosine inhibits the priming activation and proliferation of T cells by reducing the expression of effector molecules on T cells (100, 101). In patients with head and neck squamous cell carcinoma, CD73 expression on PMN-MDSCs was elevated and significantly suppressed T cell proliferation (102). In NSCLC patients, transforming growth factor (TGF-β) induces CD39 and CD73 expression on MDSCs through the mTOR/HIF-1α signaling, which makes CD39+CD73-MDSCs highly enriched in tumor tissue and produces adenosine to inhibit T cell activity (103).

Recent studies, however, have found a dual role for CD73 for proliferation and survival of T cells. On the one hand, CD73 favors the expression of IL-7 receptor α chain on CD8+ T cells and their homeostatic survival; On the other hand, under conditions of antigenic stimulation, CD73 reduces the IL-2 receptor α chain and expression of the antiapoptotic molecule Bcl-2 thereby reducing CD8+ T cell survival (104). It is therefore important to note its dual effects on CD8+ T cells when designing CD73 as a target for antitumor therapy.

#### Regulation of other immune cells

Although the immunosuppressive effects of MDSCs are mainly directed against T cells, numerous studies have shown that MDSCs can also regulate immune cells such as NK cells, macrophages, DCs, and Tregs to exert tumor promoting effects.

NK cells have played an important role in antitumor immune responses by directly killing tumor cells. It was found that after co-culture with MDSCs, NK cells had decreased cytolytic capacity, IFN-γ production capacity and natural killer group 2, member D (NKG2D) expression capacity, which was related to TGF-β produced by MDSCs (105). In addition, MDSCs can also affect NK cytotoxicity by producing NO, and this mechanism has been mentioned above.

DCs and macrophages, as antigen-presenting cells, are considered key players in antitumor immune responses. Studies have shown that MDSCs and both can interact to alter the inflammatory environment in the tumor microenvironment. IL-10 produced by MDSCs impairs the antigen-presenting function of DCs and macrophages by downregulating the expression of MHC class II, CD80 and CD86 (106). Since IL-10 is a key factor in regulating IL-12 transcription, an increase in IL-10 leads to a decrease in IL-12, which promotes the transformation of macrophages into M2 phenotype (107).

Tregs, as an important component in tumor associated immunosuppression, are able to inhibit the activation and proliferation of T cells. Under the stimulation of INF-γ, MDSCs secrete TGF-β and IL-10 up regulates the expression of Foxp3, a key transcriptional regulatory protein that determines the differentiation and function of Tregs, and upregulation of Foxp3 enhances the suppressive activity of Tregs (108, 109). In addition, in a study of F-MDSCs, it was found that the suppressive effect of F-MDSCs was not mediated by Arg-1 or iNOS mechanisms but was exerted through the generation of IDO inducing effector T cells to convert into Foxp3+ Tregs and that IDO mediated depletion of tryptophan also downregulated CD3ζ of the TCR leading to blocked T cell activation (110–112).

### Non-immunological effects of MDSCs

**Figure 3:**
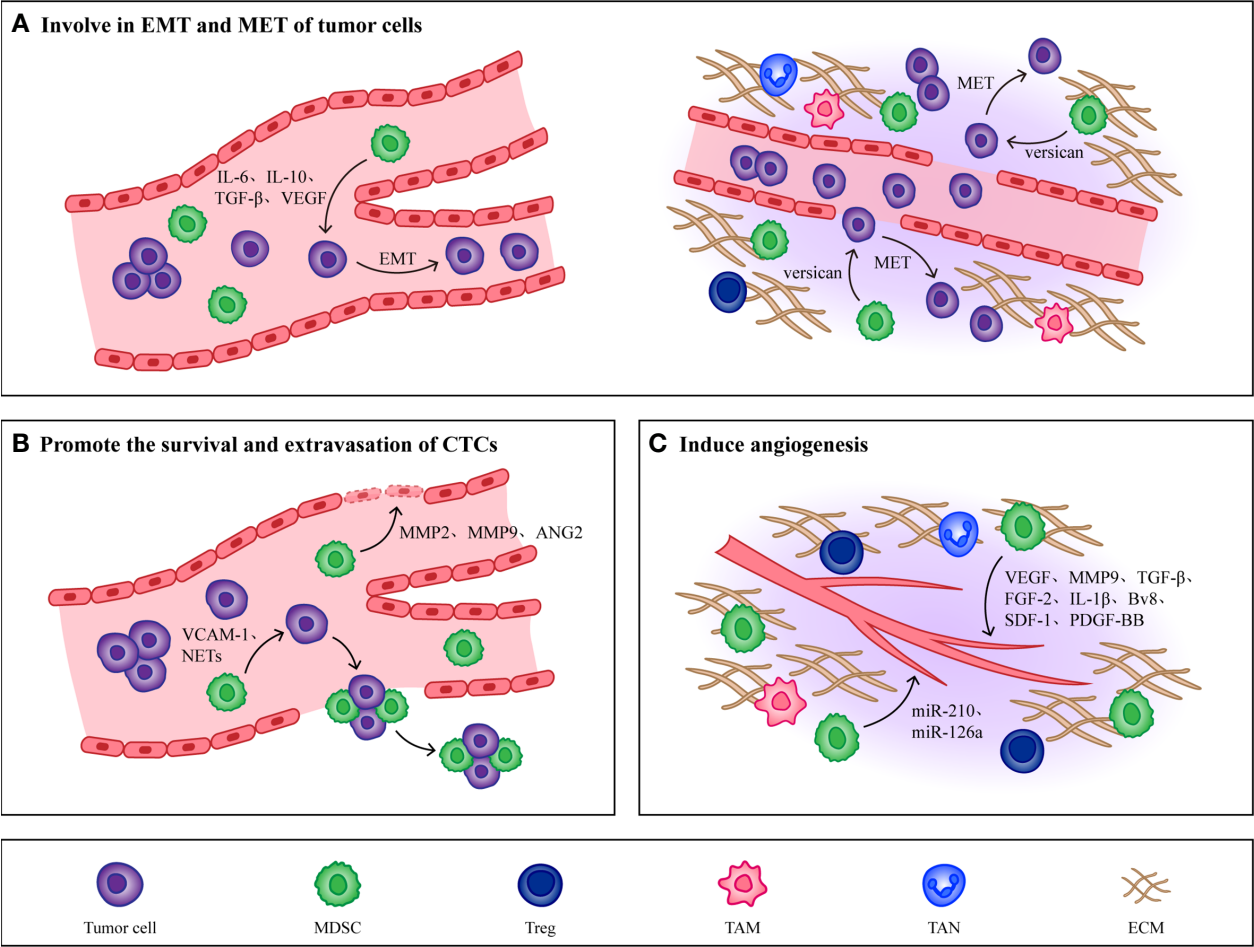
Non immunological effects of MDSCs. **(A)** MDSCs promote EMT of tumor cells by producing IL-6, IL-10, TGF-β and VEGF, which makes tumor cells obtain high migration and invasion ability. When CTCs reach the host organ, MDSCs will induce CTCs to undergo MET, restore their epithelial phenotype and promote their proliferation. **(B)** MMP2, MMP9, VEGF and Ang-2 produced by MDSCs effectively increase the permeability of blood vessels and are conducive to the extravasation of CTCs. At the same time, MDSCs combine with CTCs through VCAM-1 and NETs to form CTC/MDSC clusters, which promote the survival and extravasation of CTCs. **(C)** MDSCs promote angiogenesis in PMN by producing cytokines such as VEGF, MMP9, TGF-β and FGF-2. In addition, MDSCs derived exosomes miR-126a and miR-210 also induce angiogenesis.

In addition to establishing and maintaining an immunosuppressive microenvironment, MDSCs recruited into host organs also play a role in various steps of tumor metastasis, promoting tumor cell growth and colonization. As the best partners of tumor cells, MDSCs play many roles, such as inducing tumor cell invasion and proliferation, aiding tumor cell extravasation, and providing trophic support to tumor cells, and their mechanisms mainly include the following three points (**Figure 3**).

#### Involve in EMT and MET of tumor cells

The mesenchymal phenotype induced by EMT in primary tumors makes tumor cells lose their intercellular junctions to acquire high migratory and invasive abilities, contributing to the completion of the complex cascade of invasion metastasis (16). Cell transdifferentiation from epithelial to a mesenchymal state is mediated by key transcription factors, such as the basic helix-loop-helix factors TWIST1 and TWIST2 (113), the zinc-finger E-box-binding homeobox factors ZEB1 and ZEB2 (114), and the zinc-finger binding transcription factors SNAI1 and SNAI2 (115), which act as master regulators of cell-cell adhesion, cell polarity and motility. They repress genes associated with an epithelial phenotype and induce the expression of mesenchymal genes, ultimately leading to the cellular hallmarks of EMT.

The most direct evidence for the role of EMT in metastasis comes from the analysis of cancer patients, where it has been reported that immune cells in cancer patients regulate the induction of tumorigenic EMT by secreting cytokines and chemokines, and that tumor cells undergoing EMT also produce immunosuppressive factors and chemokines, which further induce an immunosuppressive state of the tumor microenvironment and thus promote cancer development (116, 117). Various types of cytokines produced by MDSCs, such as VEGF (118), TGF-β (119, 120), IL-6 (121, 122) and IL-10 (123) have been shown to induce EMT in tumor cells. Reported that secreted protein acidic and rich in cysteine (SPARC) from MDSCs was immunosuppressive and tumor promoting in vivo in breast cancer patients, and deletion of SPARC rendered MDSCs with reduced suppressive function and restored EMT, which illustrated that the occurrence of EMT in tumor cells may depend on SPARC secreted by MDSCs (124).

In addition to participating in the induction of EMT, MDSCs also play a role in the met process of tumor cells. Different subpopulations of MDSCs have been shown to play roles at different stages in the tumor invasion metastasis cascade. For example, induction of EMT by M-MDSCs promotes tumor invasion from the primary site to distant site, whereas induction of MET by PMN-MDSCs promotes tumor cell proliferation to support metastatic growth. This illustrates that the regulation of tumor phenotype by MDSCs changes with tumor progression (125). In addition, MDSCs also stimulate MET in tumor cells by secreting versican to attenuate phospho-Smad2 levels (126). In summary, because tumor metastasis is a multistep process and various cancers have different classification criteria, tumor location, tumor type, and tumor stage will all be important to consider in studies on the effects of MDSCs on EMT and MET.

#### Promote the survival and extravasation of CTCs

After CTCs detach from the primary tumor into the blood vessels, the mechanical and shear forces present inside the vessels will adversely affect CTCs survival as well as metastasis (127). In addition to the ability of platelets, leukocytes, and macrophages to help CTCs escape from the distress caused by these stresses, MDSCs, because of their potent immunosuppressive properties, can also contribute to the survival of CTCs (128). In pancreatic cancer patients, researchers found a positive correlation between the number of MDSCs and the number of K-RASmut mRNA+ CTCs, which suggested that the presence of MDSCs may promote the proliferation and survival of CTCs (129). Moreover, existing studies have found that MDSCs can interact with CTCs and form CTC/MDSCs clusters to promote CTCs metastasis. CTC/MDSCs cluster promoted the survival and proliferation of CTCS and enhanced their metastatic potency by activating the ROS/Notch/Nodal signaling pathway (130). Recently, it has been reported that vascular cell adhesion molecule-1 (VCAM-1) is required for CTC/MDSCs cluster formation, thus VCAM-1 inhibition could be one of the important factors hindering CTC/MDSCs cluster formation, and developing drugs targeting VCAM-1 may reduce tumor metastasis to some extent (131).

CTCs that survive overcoming the mechanical and shear forces of blood vessels when they move closer and adhere to endothelial cells, MMP2, MMP9, VEGF and Ang-2 produced by MDSCs effectively increase vascular permeability, and upregulate E-selectin, which contributes to adhesion and extravasation of CTCs (26, 132–134). Studies have shown that the formation of neutrophil extracellular traps (NETs) can induce the intravascular coagulation cascade (cancer-related thrombosis), which contributes to the growth of primary tumors, cancer invasiveness, progression and metastasis. Through the use of cecal ligation, Cools Lartigue et al. observed the presence of lung cancer cells encapsulated in NETs in a mouse model of infection. These circulating “packages” were seeded in the liver, forming micrometastases within 48 h and secondary liver cancer 2 weeks after the cancer cell injection (135). Evidence consistent with these observations was provided by Najmeh et al. from the same group, who found a significant association between upregulation of β1-integrin and NET-related entrapment of circulating lung carcinoma cells, further facilitating metastasis formation and cancer spread (136). Theoretically, NETs may play an anti-tumor role by directly killing tumor cells or activating the immune system, but it has been proved that due to partial vascular obstruction and the coagulation microenvironment around NETs, circulating tumor cells can be captured. At the same time, NETs damage endothelial cells and promote the adhesion and extravasation of CTCs.

In recent years, studies have found that PMN-MDSCs are also able to induce the formation of NETs. Alfaro et al. found that IL-8 induced PMN MDSCs to form NETs in the process of studying whether IL-8 secreted by tumors could recruit MDSCs, and observed that tumor cells were captured after NETs were formed (137). In a mouse model of lung metastatic cancer, C5a was shown to induce the expression of high mobility group box 1 (HMGB1) receptors TLR4 and rage in PMN-MDSCs, while the formation of NETs was in turn dependent on HMGB1 produced by cancer cells (138). Thus, CTC/PMN-MDSCs cluster could form NETs under the induction of complement C5a (139). Therefore, hindering the formation of NETs may affect the extravasation of CTCs and thus hinder tumor metastasis, however the presence of MDSCs can directly increase vascular permeability to enhance the extravasation of CTCs. So, the effects of MDSCs and other cytokines on CTCs need to be taken into account when developing therapies that target NETs.

#### Induce angiogenesis

In order for CTCs to colonize the PMN efficiently, PMN would generate new blood vessels to provide nutrients for CTCs to proliferate (24). MDSCs have been shown to induce the PMN angiogenesis through a variety of mechanisms, the most prominent of which is that secretion of VEGFA by MDSCs promotes neovascularization via JAK2/STAT3 signaling (140). In addition to VEGF, other cytokines derived from MDSCs promote tumor angiogenesis, such as MMP9 (141), TGF-β (142), fibroblast growth factor-2 (FGF-2) (143), IL-1β (143), bombina variegata peptide 8 (Bv8) (144), stromal cell-derived factor-1 (SDF-1) (145) and so on. Recently researchers showed that MDSCs expressed high levels of platelet-derived growth factor-BB (PDGF-BB), and they found that this not only promoted tumor angiogenesis but also increased tumor cell metastatic growth (146). In addition, MDSCs derived exosomes also contribute to tumor angiogenesis. MiR-126a released by MDSCs induced tumor angiogenesis and enhanced CTCs adhesion to endothelial cells, which promoted tumor cell metastasis (147). Under hypoxic conditions, HIF-1α upregulates MDSCs derived exosomal miR-210, which not only enhances the function of MDSCs by increasing Arg-1 activity and producing NO, but also regulates endothelial cell activation to induce tumor angiogenesis (148, 149).

## Application of MDSC in PMN detection and therapy

Early detection of the PMN formation during the course of cancer treatment helps to optimize treatment regimens and improve patient prognosis. Although there is no clinical technique at this stage available to directly visualize the dynamic process of tumor metastasis, the detection of certain specific immune cells or associated molecules in the PMN by radiological imaging offers a viable option to detect the PMN formation. In 2012, Shokeen and others used PET/CT to image very late antigen-4 (VLA-4, also known as α4β1 integrin) expressing MDSCs in PMN (150). Li and others evaluated the effectiveness of CT images in quantifying the microenvironment changes in the premetastatic lung. Their results suggested that changes in the lung microenvironment can be identified by CT before primary breast cancer lung metastasis (151). Based on the role of S100A8/A9 in promoting MDSCs accumulation and inducing the PMN, researchers reported the application of antibody-based SPECT for detection of S100A8/A9 in vivo as an imaging marker for pre-metastatic tissue priming (152). In addition to S100A8/A9, other MDSCs derived exosomes, such as miR-126a and miR-210 mentioned earlier, have also been implicated in PMN formation. But there are no effective tracers for these molecules, and their distribution profile in the pre metastatic microenvironment is unclear. Therefore, further studies are needed to test MDSCs derived exosomes as an indicator of the PMN.

**Figure 4:**
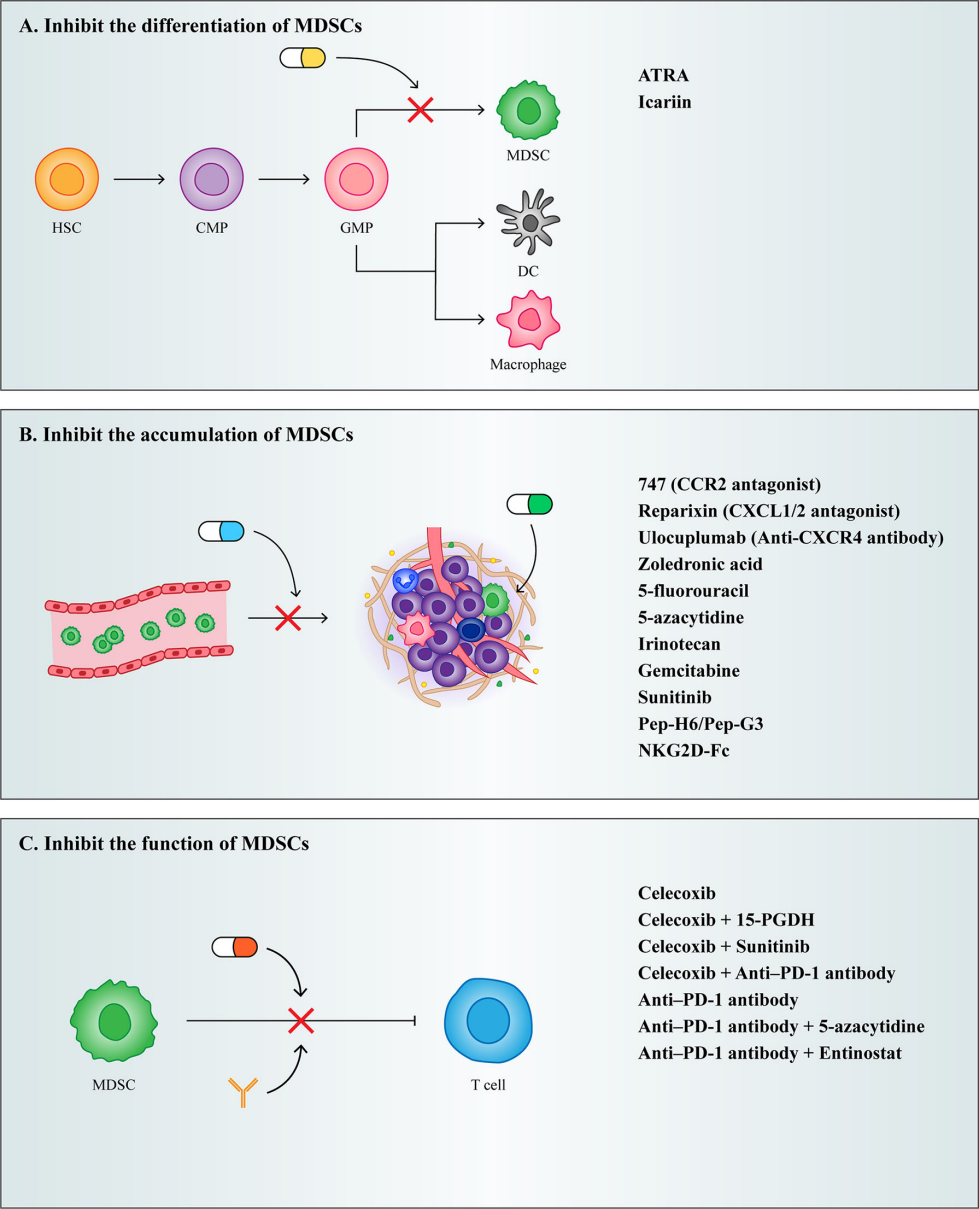
Treatment strategies for MDSCs. **(A)** Inhibit the differentiation of MDSCs. **(B)** Inhibit the accumulation of MDSCs. **(C)** Inhibit the function of MDSCs.

With the development of cancer treatment technology, the rise of immunotherapy exemplifies the shift in cancer treatment from predominantly tumor suppressor to predominantly PMN modulating. Although targeted therapies to PMN can potentially inhibit tumor metastasis, it is difficult to target drugs to PMN owing to the aberrant tumor microenvironment generation that results from increased vascular permeability in PMN (33). Currently, clinical therapeutic strategies targeting PMN have shifted to specifically targeting PMN composition to suppress tumor metastasis. As one of the most important cells driving PMN formation, MDSCs can serve as an important target for inhibiting tumor metastasis. Currently, therapeutic strategies targeting MDSCs both inhibit MDSCs differentiation and accumulation as well as their function (**Figure 4**).

### Inhibit the differentiation of MDSCs

As described previously, IMC differentiates into mature granulocytes, macrophages, and DCs under normal circumstances, and when this process is blocked by certain cytokines, it results in the accumulation of MDSCs. Therefore, the induction of MDSCs into mature myeloid cells may be a potential strategy for the treatment of cancer.

Vitamin A has been shown to induce MDSCs differentiation. Studies have shown that all trans retinoic acid (ATRA), a metabolite intermediate of vitamin A, induces the rapid differentiation of MDSCs into macrophages and DCs by activating the ERK1/2 pathway, which reduces the suppressive function of MDSCs (153). However, it has been shown that ATRA also promotes Foxp3 expression and enhances the function of CD4+ Tregs. Therefore, while using ATRA to target MDSCs for therapy, it is important to note that ATRA may induce the generation of Tregs and thereby promote tumor development (154).

Furthermore, Chinese scholars have proved that Icariin, the main active component of Herba Epimedii, can mediate anti-inflammatory function. Here, they showed that treatment of tumor bearing mice using icariin derivatives could significantly decrease the percentage of MDSCs and their differentiation into macrophages and DCs (155).

### Inhibit the accumulation of MDSCs

Because MDSCs express high levels of chemokine receptors and are recruited to PMN by tumor derived chemokines, preventing the recruitment of MDSCs could be achieved by targeting chemokine receptors on MDSCs. Chemokine receptor antagonists in multiple clinical trials, such as the CCR2 antagonist 747 (156), the CXCR1/2 antagonist Reparixin (157), and the CXCR4 antibody Ulocuplumab (158), have all been shown to reduce the infiltration of MDSCs in tumors, ultimately reducing their metastatic potential.

In addition to chemokine antagonists, aminobisphosphonates have also been shown to inhibit the recruitment of MDSCs. Aminobisphosphonates reduce MMP9 expression and the number of macrophages in the tumor stroma and decrease MDSCs infiltration in bone marrow and peripheral blood by decreasing serum pro-MMP9 and VEGF (159). In a mouse model of pancreatic cancer, the use of zoledronic acid reduced MDSCs accumulation and slowed tumor growth (160).

Depleting MDSCs recruited into PMN is also an effective therapeutic strategy. Low dose chemotherapeutic drugs have been shown to effectively eliminate MDSCs in tumor bearing mice. Given to tumor bearing mice, a significant decrease in the number of MDSCs can be observed after treatment with various chemotherapeutic agents, such as 5-fluorouracil (161), irinotecan (162), gemcitabine (163), and sunitinib (164). In addition to chemotherapeutic agents, researchers have developed fusion proteins capable of targeting MDSCs. Qin developed a novel therapeutic peptide-Fc fusion protein that specifically depletes MDSCs without affecting other pro-inflammatory classes of immune cells (165). Subsequently, Feng synthesized an NKG2D-Fc fusion protein, which reduced infiltrating MDSCs in tumors by binding to NKG2D expressing MDSCs (166).

### Inhibit the function of MDSCs

Because MDSCs play an important role in the tumor microenvironment and PMN due to their powerful immunosuppressive function, alleviating the immunosuppressive mechanisms of MDSCs has become a major therapeutic target to re-establish T-cell activity.

COX2 inhibitors have been reported to act as immunosuppressive agents to improve tumor patient survival. COX2 induces ROS production in MDSCs through the production of prostaglandin E2 (PGE2), and disruption of COX2/PGE2 signaling contributes to the attenuation of the suppressive function of MDSCs. In a murine mesothelioma model, the use of a single COX2 inhibitor, celecoxib, was able to reduce MDSCs levels and reverse T cell function (167). It was subsequently shown that treatment with celecoxib in combination with 15-hydroxyprostaglandin dehydrogenase (15-PGDH) (168), sunitinib (169), and PD-1 (170) in a murine metastatic tumor model more effectively reduced MDSCs levels as well as increased T cell numbers. Therefore, multiple drug combination therapy may be used selectively on therapeutic strategies targeting MDSCs for the patient’s own condition.

In the last two years, researchers found that triggering receptor expressed on myeloid cells (TREMs) has emerged as a potent regulator of innate immune responses, and observed that TREM1 was expressed in M-MDSCs and PMN-MDSCs, but TREM2 was expressed only in the M-MDSCs. They performed contrasting experiments by overexpressing and underexpressing TREM1 and observed worse prognosis in cancer patients with increased levels of TREM1 (171). Martina’s experiments suggest that TREM2 deficiency may promote increased T cell activation and may enhance responsiveness to anti-PD-1 checkpoint blockade, and treatment with anti-TREM2 mAb curbed tumor growth and fostered regression when combined with anti-PD-1 (172). Therefore, TREMs may be a useful biomarker of tumor progression, and developing TREMs inhibitors might be able to effectively combat tumor development.

Nowadays, combining MDSCs targeted therapy and immunotherapy has emerged as a new therapeutic strategy in improving the therapeutic efficacy of targeted MDSCs. The investigators found that mice still had large primary tumors and metastases that were not eliminated after treatment with a single anti-PD-1, whereas when anti-PD-1 was combined with 5-azacytidine and entinostat, both primary tumors in mice were eliminated and no metastases appeared (173). This indicates that immunotherapy combined with MDSCs targeted therapy is more effective in improving tumor progression and improving patient survival.

## Conclusion

PMN formation reflects the dynamic relationship between tumor cells and the microenvironment of metastatic sites. Exploring PMN formation as well as the understanding of PMN biology has largely relied on mouse models of lung metastases. Thus, more clinical studies are needed, and more direct assays should be developed on the basis of existing techniques for PMN detection in order to observe the dynamic course of tumor metastasis.

In PMN formation, MDSCs play an important immunosuppressive role because of their specific heterogeneity. PMN-MDSCs suppress T cell function mainly through the production of ROS, and because ROS are very labile, PMN-MDSCs need to be in close contact with T cells to exert suppressive effects. In contrast, M-MDSCs suppress T cell activation by producing large amounts of iNOS, Arg-1, and some immunosuppressive factors. The half-life of iNOS, Arg-1, is much longer compared to ROS, so M-MDSCs need not come into close contact with T cells only to exert their suppressive effect. This illustrates the higher inhibitory activity of M-MDSCs than PMN-MDSCs (84, 174–177). Compared with the first two subsets of MDSCs, aspects such as the origin and function of e-MDSCs are generally less well recognized. It has been suggested that e-MDSCs do not have an inhibitory effect on the proliferation of T cells (178), and conversely, it has been shown that e-MDSCs have a very low inhibitory effect (179). Therefore, whether e-MDSCs can be considered as M-MDSCs, PMN-MDSCs such functional MDSCs is still controversial and needs further exploration. In contrast, F-MDSCs exert immunosuppressive effects by inducing the conversion of effector T cells into Tregs through the production of IDO, as described previously.

In addition to their own immunosuppressive functions, MDSCs have been shown to play important roles in vascular leakage, ECM remodeling, angiogenesis, and so on. Based on the role of MDSCs at various stages in PMN formation, researchers have developed therapeutic approaches to target MDSCs to detect the tumor metastasis situation at early stages of cancer. Although numerous studies have demonstrated the pro metastatic role of MDSCs, additional studies have shown that MDSCs can suppress tumor metastasis in some contexts. High amounts of anti-angiogenic TSP-1 are produced by MDSCs in tumors that lack metastatic potential, and knockdown of TSP-1 restores the pro tumor effects of MDSCs (180). Attention needs therefore to be paid to the effects of TSP-1 in therapies that block tumor metastasis by targeting MDSCs. Nowadays, for targeted therapy of MDSCs, it is important to focus not only on MDSCs regulatory network but also on the mutual communication between MDSCs and other cells or components, which has the potential to provide new options for cancer therapy.

## Author contributions

GY drafted the article and did literature search. WR revised the article, provided ideas on article structure, and added some content. RQ, JH and SZ modified the arrangement of the article and helped to improve the accuracy of the language. All authors contributed to the article and approved the submitted version.

## Funding

This work was supported by the National Natural Science Foundation of China (82174146) and grants from Henan Provincial Science and Technology Research Project (grant no. 212102310639), Key Scientific Research Project of Colleges and Universities in Henan Province (grant no. 19zx009).

## Conflict of Interest

The authors declare that the research was conducted in the absence of any commercial or financial relationships that could be construed as a potential conflict of interest.

## Publisher’s note

All claims expressed in this article are solely those of the authors and do not necessarily represent those of their affiliated organizations, or those of the publisher, the editors and the reviewers. Any product that may be evaluated in this article, or claim that may be made by its manufacturer, is not guaranteed or endorsed by the publisher.

## Abbreviations

Arg-1, arginase-1; BMDCs, bone marrow-derived cells; COX2, cyclooxygenase 2; CSCs, cancer stem cells; CTCs, circulating tumor cells; DCs, dendritic cells; DDR1, discoidin domain receptor 1, DDR1; ECM, extracellular matrix; e-MDSCs, early myeloid derived suppressor cells; EMT, epithelial mesenchymal transition; F-MDSCs, fibrocytic myeloid derived suppressor cells; IDO, indolamine 2, 3-dioxygenase; iNOS, inducible nitric oxide synthase; MET, mesenchymal epithelial transition; MDSCs, myeloid derived suppressor cells; M-MDSCs: monocytic myeloid derived suppressor cells; NETs, neutrophil extracellular traps; PD-1, programmed death-1; PD-L1, programmed death-ligand 1; PMN, pre-metastatic niche; PMN-MDSCs, polymorphonuclear myeloid derived suppressor cells; ROS, reactive oxygen species; TDSFs, tumor-derived secretory factors; TGF-β, transforming growth factor; Tregs, regulatory T cells; TREMs, triggering receptor expressed on myeloid cells; VEGF, vascular endothelial growth factor; ZO-1, zonula occludens 1.
